# Exploring the correlation among genetic variants, cholecystectomy and gut microbiome: A Mendelian randomization study

**DOI:** 10.1097/MD.0000000000039852

**Published:** 2024-09-27

**Authors:** Yulai Yin, Xiaoyu Zhang

**Affiliations:** a Cangzhou Central Hospital, Hebei Medical University, Cangzhou, China; b Department of Thyroid and Breast Surgery III, Cangzhou Central Hospital, Cangzhou, China.

**Keywords:** cholecystectomy, cholelithiasis, gut microbiota, instrumental variables, Mendelian randomization

## Abstract

This Mendelian randomization (MR) study aims to explore the relationship between gut microbiota and the occurrence of cholelithiasis, as well as the impact of cholecystectomy on the gut microbiota. This study leverages data on exposures and outcomes from the GWAS database, employing the inverse variance weighting (IVW) method to obtain primary causal estimates. Heterogeneity is assessed using Cochran *Q* and Rücker *Q* tests through both IVW and MR-Egger methods. Pleiotropy is evaluated using the Egger-intercept method, while sensitivity analyses are conducted via leave-one-out tests. Additionally, the *F*-statistic is calculated to assess the presence of weak instrument bias. Finally, the MR-PRESSO method is utilized to validate the findings concerning the relationship between gut microbiota and the incidence of cholelithiasis, as well as the impact of cholecystectomy on gut microbiota composition. The genera Butyricicoccus (ID: 2055), Solibacillus (ID: 11348), Anaerotruncus (ID: 2054), Allisonella (ID: 2174), and Howardella (ID: 2000) have been found to decrease the genetically predicted probability of cholelithiasis. Reverse MR analysis indicates that the occurrence of cholelithiasis reduces the levels of gut microbiota such as Blautia (ID: 1992), Anaerofilum (ID: 2053), Howardella (ID: 2000), Butyricicoccus (ID: 2055), Solibacillus (ID: 11348), Allisonella (ID: 2174), Anaerotruncus (ID: 2054), and Firmicutes (ID: 1672). Additionally, the genera Odoribacter (ID: 952), and Holdemanella (ID: 2157) increase the genetically predicted risk of cholecystectomy. Reverse MR results show that post-cholecystectomy reduces the levels of gut microbiota such as Blautia (ID: 1992), Butyricicoccus (ID: 2055), Alistipes (ID: 11296), Oxalobacteraceae (ID: 2966), and Ruminococcaceae UCG010 (ID: 11367). Conversely, post-cholecystectomy increases the levels of gut microbiota such as Odoribacter (ID: 952), an unknown family (ID: 1000001214), an unknown genus (ID: 1000001215), Aeromonadales (ID: 1591), Holdemanella (ID: 2157), Phascolarctobacteria (ID: 1589), and Eggerthella (ID: 819). All study results show no horizontal pleiotropy, and the MR-PRESSO validation results are consistent with the MR analysis findings. This study elucidates the relationship between gut microbiota and the occurrence of cholelithiasis, as well as the impact of cholecystectomy on the gut microbiota. These findings have clinical significance for diagnosing disease onset and understanding digestive function changes following gallbladder removal, providing theoretical support for further investigation into the molecular mechanisms underlying cholelithiasis.

## 1. Introduction

Cholelithiasis^[1–4]^ refers to the formation of solid deposits in the gallbladder or bile ducts, primarily composed of cholesterol and/or bilirubin. The formation of these stones is typically associated with various factors, including cholesterol metabolism imbalance, disproportionate levels of bile salts and cholesterol, and dysfunctional gallbladder motility. Cholelithiasis can be asymptomatic or cause biliary colic, a pain that usually occurs after meals, particularly following the intake of high-fat foods. Complications from gallstones include acute cholecystitis, cholangitis, obstructive jaundice, and pancreatitis, which may require urgent medical intervention. A meta-analysis^[5]^ reported a prevalence of cholelithiasis at 6.1% (95% CI: 5.6–6.5), with higher prevalence in females versus males (7.6% vs 5.4%), South America versus Asia (11.2% vs 5.1%), and middle-high-income countries versus high-income countries (8.9% vs 4.0%), and an increase with age. Cholecystectomy,^[6]^ or gallbladder removal surgery, is a common procedure primarily used to treat gallbladder diseases, especially cholelithiasis. This surgery can be performed as open surgery or laparoscopically, with the latter being preferred due to faster recovery, less pain, shorter hospital stays, and smaller scars, making it the mainstream method both domestically and internationally.

Gut microbiota^[7–9]^ is increasingly recognized as a significant environmental factor influencing human health. It has been identified as both a risk and preventive factor for various diseases and has shown causal associations with multiple conditions. Observational studies have noted that different compositions of gut microbiota are associated with varying risks of developing gallstones. Several studies have suggested that gut microbiota might influence bile metabolism and thus affect gallstone formation. For instance, Wang et al^[10]^ found that Clostridium spp. act as protective factors against gallstone formation, while Ye et al ^[11]^ identified Lactobacillus spp. as protective. Additionally, most scholars consider Firmicutes to be a risk factor for gallstone formation. Similarly, there is no consensus from cross-sectional and observational studies on changes in gut microbiota post-cholecystectomy. Previous observational studies exploring the relationship between gut microbiota and cholelithiasis have been significantly influenced by confounding factors such as age, environment, diet, and lifestyle, resulting in divergent conclusions. The lack of consistent and rigorous conclusions regarding changes in gut microbiota post-cholecystectomy is also due to the challenges of long-term follow-up and the influence of confounding factors like environment and diet. The presence of confounding factors limits the inference of the association between gut microbiota and gallbladder outcomes. Basic experimental exploration of gut microbiota and disease development involves long follow-up periods, ethical considerations for animal experiments, and high financial and resource costs. Mendelian randomization (MR), based on single nucleotide polymorphisms (SNPs), infers causal relationships between exposures and disease outcomes through genetic variations. In MR studies, genetic variants associated with phenotypes are used as IVs to infer causal relationships between exposure and outcome. Genetic variation follows the random segregation of alleles from parents to offspring and is determined at conception by genetic variation, thus minimizing confounding in traditional observational studies.

Therefore, it is essential to conduct a MR study to explore 2 critical scientific questions: the relationship between gut microbiota and the occurrence of cholelithiasis, and the changes in gut microbiota following cholecystectomy.

## 2. Materials and methods

### 2.1. Exposure data

We selected SNPs associated with the composition of the human gut microbiome from the GWAS dataset provided by the International MiBioGen Consortium. This dataset is a large-scale, multi-ethnic GWAS covering 24 cohorts from the USA, Canada, Israel, Korea, Germany, Denmark, Netherlands, Belgium, Sweden, Finland, and the UK, with a total of 18,340 participants. The 16S rRNA gene sequencing profiles and genotype data of these participants were used to study the association between human genetic variation on autosomes and the gut microbiome. The dataset includes 211 taxa, comprising 131 genera, 35 families, 20 orders, 16 classes, and 9 phyla.

### 2.2. Outcome data

For cholelithiasis, we used summary statistics from a GWAS, including 7895 cases and 476,703 controls (https://gwas.mrcieu.ac.uk/datasets/ebi-a-GCST90038629/). Data related to cholecystectomy were obtained from a GWAS of 18,319 cases and 444,614 controls of European ancestry (https://gwas.mrcieu.ac.uk/datasets/ukb-b-6235/).

### 2.3. Selection of instrumental variables

In MR studies, to ensure valid and reliable conclusions, the selection of instrumental variables (IVs) must satisfy the following 3 assumptions: genetic variants must be strongly associated with the exposure; they must influence the outcome solely through the exposure, not directly or via other pathways; they must not be related to any confounding factors of the exposure-outcome relationship.

To minimize the effects of linkage disequilibrium^[12,13]^ (LD), we selected SNPs meeting the recognized genome-wide significance thresholds (*P* < 1 × 10^−5^ for exposures; *P* < 5 × 10^−8^ for outcomes, *r*^2^ ≤ 0.001, meeting Hardy–Weinberg equilibrium (H-W), genetic distance < 10,000 kb) as IVs. The *F*-statistic was calculated to ensure that IVs with *F* > 10 were included to avoid bias from weak instruments.

### 2.4. Data availability

Exposure-related data can be found in Supplementary File 1, Supplemental Digital Content, http://links.lww.com/MD/N651; outcome-related data can be accessed and downloaded from the aforementioned GWAS database links.

### 2.5. Statistical analysis

We used the inverse variance weighting (IVW) MR analysis as the primary method, performing heterogeneity tests with IVW and MR-Egger methods using Cochran *Q* and Rücker *Q* tests, pleiotropy assessment using Egger-intercept method, and sensitivity analysis via leave-one-out approach.

To strengthen the evidence, we conducted an independent MR study using the PhenoScanner database,^[14]^ excluding pleiotropic SNPs related to exposure-outcome confounding factors. Effect estimates are expressed as odds ratios (OR) with 95% confidence intervals (CI).

Given multiple testing, associations with *P*-values below the Bonferroni correction threshold α = 0.05/211 = 0.00237 were considered statistically significant, while those with *P*-values ≥ .00237 and <.05 were deemed suggestively significant. All analyses were performed using R software (version 4.3.2), with MR analysis conducted using the “TwoSampleMR” package. Figures were plotted using R packages such as “ggplot2,” “plyr,” “dplyr,” “purrr,” “readr,” “pacman,” “ieugwasr,” and “MRInstruments.” Reporting follows the STROBE-MR^[15,16]^ (strengthening the reporting of observational studies in epidemiology – MR) guidelines.

### 2.6. Ethical statement

This study was conducted using publicly available GWAS databases. All original studies had obtained ethical approval.

## 3. Results

### 3.1 Mendelian randomization analysis

**Figure 1.**
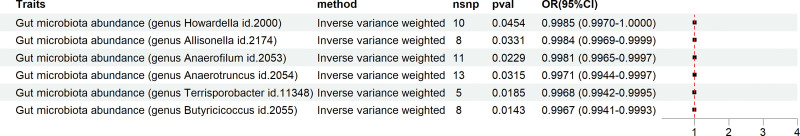
This figure demonstrates the Mendelian randomization (MR) analysis using the inverse variance weighting (IVW) method as the primary approach. “Traits” represent the corresponding gut microbiota categories. “Method” indicates the IVW method used for MR analysis. “Nsnp” denotes the number of single nucleotide polymorphisms (SNPs) used as instrumental variables (IVs). “Pval” refers to the corresponding *P*-value, and “odds ratios (OR) (95% confidence intervals [CI])” represents the estimated odds ratio and 95% confidence interval. On the right side of the figure is a forest plot with a red reference line at 1. Segments to the left of the red line indicate a negative association between exposure and outcome, while those to the right suggest a positive association. Segments crossing the red line imply no clear evidence of an association between exposure and outcome. If no segment is displayed, it means the OR value and 95% CI are either extremely close to 0 or far >1. The specific positive or negative associations should be interpreted based on the OR (95% CI) values provided in the table.

**Figure 2.**
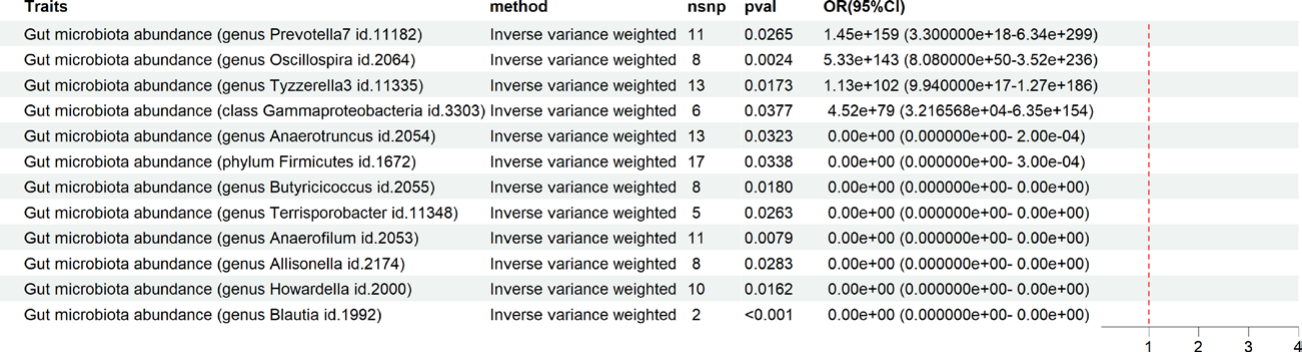
This figure illustrates the results of the MR analysis using the IVW method as the primary approach. “Traits” denotes the corresponding categories of gut microbiota. “Method” indicates that the IVW method was employed for the MR analysis. “Nsnp” represents the number of SNPs used as IVs. “Pval” refers to the corresponding *P*-value, while “OR (95% CI)” denotes the estimated odds ratio and its 95% confidence interval. On the right side of the figure is a forest plot with a red reference line at 1. Segments to the left of the red line indicate a negative association between exposure and outcome, while those to the right suggest a positive association. Segments crossing the red line imply no clear evidence of an association between exposure and outcome. If no segment is displayed, it signifies that the OR value and its 95% CI are either extremely close to 0 or significantly >1. The specific positive or negative associations should be interpreted based on the OR (95% CI) values provided in the table.

Using the IVW method as the primary analytical approach,^[17,18]^ the results are as follows. Initially, a bidirectional MR analysis was conducted with 211 types of gut microbiota as exposures and cholelithiasis as the outcome. The findings indicate that the genera Butyricicoccus (ID: 2055), Solibacillus (ID: 11348), Anaerotruncus (ID: 2054), Allisonella (ID: 2174), and Howardella (ID: 2000) can reduce the genetically predicted probability of developing cholelithiasis (Fig. 1). Conversely, reverse MR analysis revealed that the occurrence of cholelithiasis decreases the levels of gut microbiota such as Blautia (ID: 1992), Anaerofilum (ID: 2053), Howardella (ID: 2000), Butyricicoccus (ID: 2055), Solibacillus (ID: 11348), Allisonella (ID: 2174), Anaerotruncus (ID: 2054), and Firmicutes (ID: 1672). Moreover, cholelithiasis was found to increase the levels of gut microbiota such as Oscillospira (ID: 2064), Tezzerella 3 (ID: 11335), Prevotella 7 (ID: 11182), and Gammaproteobacteria (ID: 3303) (Fig. 2).

#### 3.1.1. *Bidirectional* Mendelian randomization *analysis for cholecystectomy*

**Figure 3.**
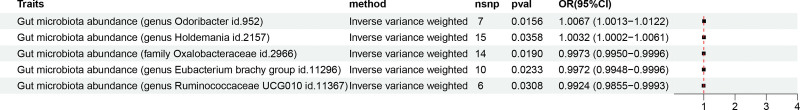
This figure illustrates the results of the MR analysis using the IVW method as the primary approach. “Traits” denotes the corresponding categories of gut microbiota. “Method” indicates that the IVW method was employed for the MR analysis. “Nsnp” represents the number of SNPs used as IVs. “Pval” refers to the corresponding *P*-value, while “OR (95% CI)” denotes the estimated odds ratio and its 95% confidence interval. On the right side of the figure is a forest plot with a red reference line at 1. Segments to the left of the red line indicate a negative association between exposure and outcome, while those to the right suggest a positive association. Segments crossing the red line imply no clear evidence of an association between exposure and outcome. If no segment is displayed, it signifies that the OR value and its 95% CI are either extremely close to 0 or significantly >1. The specific positive or negative associations should be interpreted based on the OR (95% CI) values provided in the table.

: Subsequently, a bidirectional MR analysis was performed with 211 types of gut microbiota as exposures and cholecystectomy as the outcome. The results indicated that the genera Odoribacter (ID: 952) and Holdemanella (ID: 2157) increased the genetically predicted risk of cholecystectomy, whereas the families Oxalobacteraceae (ID: 2966), Alistipes (ID: 11296), and Ruminococcaceae UCG010 (ID: 11367) decreased the genetically predicted risk of cholecystectomy (Fig. 3).

**Figure 4.**
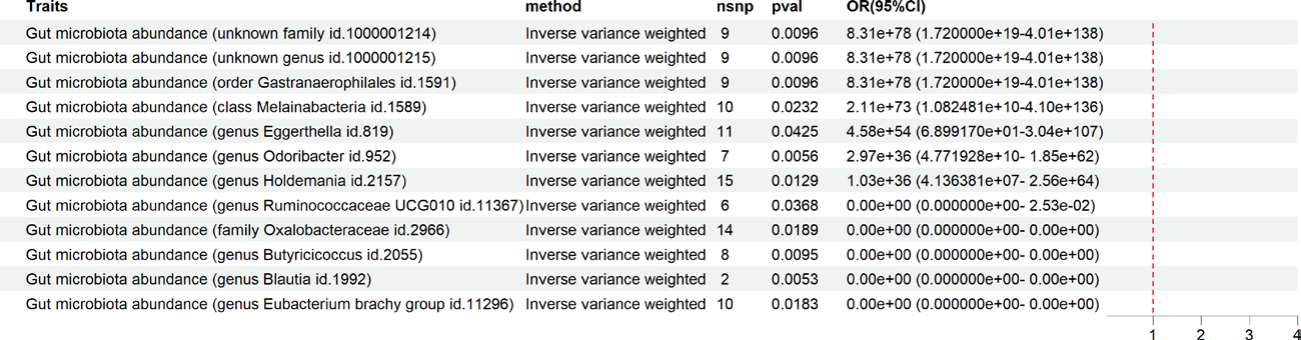
This figure illustrates the results of the MR analysis using the IVW method as the primary approach. “Traits” denotes the corresponding categories of gut microbiota. “Method” indicates that the IVW method was employed for the MR analysis. “Nsnp” represents the number of SNPs used as IVs. “Pval” refers to the corresponding *P*-value, while “OR (95% CI)” denotes the estimated odds ratio and its 95% confidence interval. On the right side of the figure is a forest plot with a red reference line at 1. Segments to the left of the red line indicate a negative association between exposure and outcome, while those to the right suggest a positive association. Segments crossing the red line imply no clear evidence of an association between exposure and outcome. If no segment is displayed, it signifies that the OR value and its 95% CI are either extremely close to 0 or significantly >1. The specific positive or negative associations should be interpreted based on the OR (95% CI) values provided in the table.

Reverse MR analysis revealed that post-cholecystectomy, the levels of gut microbiota such as Blautia (ID: 1992), Butyricicoccus (ID: 2055), Alistipes (ID: 11296), Oxalobacteraceae (ID: 2966), and Ruminococcaceae UCG010 (ID: 11367) decreased. Conversely, post-cholecystectomy increased the levels of gut microbiota such as Odoribacter (ID: 952), an unknown family (ID: 1000001214), an unknown genus (ID: 1000001215), Aeromonadales (ID: 1591), Holdemanella (ID: 2157), Phascolarctobacteria (ID: 1589), and Eggerthella (ID: 819) (Fig. 4).

### 3.2. Sensitivity analysis

The heterogeneity tests using the IVW method and MR-Egger method include Cochran *Q* and Rücker *Q* tests, where *P* > .05 indicates the presence of heterogeneity. In cases of detected heterogeneity, the MR-PRESSO method is employed, selecting 1000 distributions to remove outliers. For horizontal pleiotropy assessment using the Egger-intercept method, *P* > .05 indicates the absence of horizontal pleiotropy. The results of heterogeneity and horizontal pleiotropy tests before outlier removal are presented in Supplementary File 2, Supplemental Digital Content, http://links.lww.com/MD/N651. The MR analysis results presented in this study show no evidence of horizontal pleiotropy, and MR-PRESSO method validation maintains statistical significance, as detailed in Supplementary File 2, Supplemental Digital Content, http://links.lww.com/MD/N651.

## 4. Discussion

This study employs a bidirectional 2-sample MR approach to investigate 2 critical issues: the causal relationship between gut microbiota and the incidence of gallstone disease, and the impact of cholecystectomy on the composition of gut microbiota, preliminarily revealing the association between gut microbiota and related gallbladder outcomes. The study elucidates the bidirectional causal relationship between gut microbiota and both gallstone disease and cholecystectomy. This understanding enhances our comprehension of the role of gut microbiota in digestive diseases, contributing significantly to the field of gut microbiota and digestive disease research. Furthermore, this exploration offers new perspectives for the prevention and management of gallstone disease and post-cholecystectomy care, recognizing the crucial role of gut microbiota. From a public health standpoint, this elucidation provides scientific evidence for formulating public health policies aimed at preventing digestive diseases and reducing the global burden of gallstones. More importantly, this study bridges the gap between microbiology and digestive diseases, revealing the critical role of gut microbiota in digestive health and promoting interdisciplinary research between the 2 fields. Clinically, the findings offer new approaches for preventing and treating gallstone disease and post-cholecystectomy recovery through the modulation of gut microbiota, potentially leading to novel therapeutic strategies and interventions. For the population, a deeper understanding of the relationship between gut microbiota, gallstone disease, and cholecystectomy contributes to personalized health management and supports the advancement of precision medicine.

Current understanding of the causal relationship between gut microbiota and cholelithiasis, and the impact of cholecystectomy on the gut microbiota composition, remains inconclusive, with a dearth of high-level evidence-based studies to delineate these associations. A study by Wang et al^[19]^ reports a significant reduction in intestinal bacterial diversity and the abundance of certain phyla, particularly Firmicutes, in individuals with gallstones. This research also confirms that dysbiosis affects bile acid and cholesterol metabolism, contributing to gallstone formation, aligning closely with our findings. Furthermore, a murine model^[8]^ demonstrated that desulfovibrionaceae promote gallstone formation by altering bile acid metabolism. The specific mechanisms involve increased production of secondary bile acids and enhanced hydrophobicity in the cecum, facilitating intestinal cholesterol absorption. The metabolic byproducts of desulfovibrionaceae, including increased H2S, were shown to induce liver FXR and suppress CYP7A1 expression, while inducing hepatic expression of the cholesterol transporter Abcg5/g8 to promote biliary cholesterol secretion. All these factors collectively contribute to gallstone formation. It is possible that the absence of a significant causal link between desulfovibrionaceae and cholelithiasis in our study could be attributed to differences in the gut microbiota composition between humans and mice. In contrast to the unidirectional 2-sample MR study conducted by Liu et al,^[20]^ this study did not observe any microbial taxa promoting gallstone formation, including those from the Firmicutes phylum, which are widely recognized by most researchers. This discrepancy may be attributed to the differences in the selection of the 211 gut microbiota species in this study compared to those chosen by other researchers. Further investigation into the comparative toxicogenomics database (https://ctdbase.org/) has identified 4 genes – HMGCR, ABCG8, CCK, and GNAS – as having direct evidence of associations with gallstone formation. Additionally, the database reveals that pathways KEGG:hsa04976 and REACT:R-HSA-1430728, which involve 3 or more related genes, modulate gallstone formation through their effects on bile secretion and metabolism. From a biomedical perspective, it is suggested that the gut microbiome may influence gallstone development by altering the expression of these genes, thereby impacting bile secretion and metabolic processes. A meta-analysis of cross-sectional studies by Fusheng Xu et al^[21]^ investigated the differences in gut microbiota composition between cholecystectomy patients and healthy individuals. There is considerable controversy; evidence from 3 out of 4 studies suggests that genera such as Blautia and Gastrococci are elevated in the cholecystectomy group compared to the healthy controls. However, our findings concur with 1 out of 4 studies, suggesting a significant decrease in these genera post-cholecystectomy, with substantial OR. A consistent observation is that changes in bile metabolism post-cholecystectomy influence the stability and balance of the intestinal microbiota, thereby increasing the likelihood of intestinal inflammation. Regarding the potential mechanisms linking gut microbiota and gallbladder function, variations in the composition of the gut microbiota may alter the digestive speeds of food in different segments of the intestine, such as the jejunum, ileum, and colon. This variability can influence the rate of gastric emptying and, indirectly, bile secretion, which in turn can either promote or inhibit the formation of gallstones. Similarly, following cholecystectomy, bile is synthesized and secreted directly by the liver, bypassing the gallbladder’s role in concentrating and storing bile. The resultant changes in bile concentration indirectly affect the composition of the gut microbiota, which may lead to an increased likelihood of intestinal diseases. This intricate interplay highlights the critical role of microbial populations in gastrointestinal pathophysiology and their influence on hepatic functions.

This study presents several strengths: Firstly, it employs a bidirectional 2-sample MR approach,^[12,13,22,23]^ which confirms the robustness of the results by showing consistent causal associations. Secondly, it incorporates heterogeneity tests and horizontal pleiotropy assessments to validate the scientific integrity of the findings, using MR-PRESSO to further ensure the robustness of the results. Thirdly, the study extensively covers 211 taxa of gut microbiota, exploring a comprehensive range of genera, families, orders, classes, and phyla to deepen our understanding of the bidirectional causality between gut microbiota and gallstone diseases, as well as the effects of cholecystectomy. Fourthly, the reliability of the findings is demonstrated through forest plots that visually present the results of IVW MR analyses. Lastly, the data on gut microbiota and gallstone disease is based on the whole human genome, offering broad applicability and generalizability of the findings.

However, the study also has limitations: Firstly, it is confined to a European population for cholecystectomy subjects, which may limit the generalizability of conclusions drawn about post-cholecystectomy outcomes. Secondly, the range of gut microbiota taxa included in the study is not exhaustive, indicating the need for further exploration. Thirdly, the lack of temporal data post-cholecystectomy prevents an assessment of changes in gut microbiota composition over the same time points following surgery. Fourthly, the use of the GWAS database limits the distinction between cholecystolithiasis and choledocholithiasis under the general term “gallstone disease,” hindering more specific conclusions. Finally, due to limited resources and restricted data access, this study lacks validation from additional databases, which could strengthen the findings.

Therefore, future research should focus on observational studies and randomized controlled trials involving diverse populations, large-scale, multicenter designs, and long follow-up periods to validate the findings of this study and enhance the adaptability of the conclusions. At the basic medical level, further exploration of the specific mechanisms by which gut microbiota influences gallstone disease and post-cholecystectomy recovery is warranted to uncover their potential biological roles and pathways. From a translational medicine perspective, integrating the study’s findings into clinical practice by gradually applying gut microbiota modulation techniques for the prevention of gallstone disease and the management of post-cholecystectomy recovery is essential to verify the clinical efficacy and feasibility of the research results.

## 5. Conclusion

This study elucidates the relationship between gut microbiota and the occurrence of cholelithiasis, as well as the impact of cholecystectomy on the gut microbiota. These findings hold clinical significance for detecting disease onset and understanding changes in digestive function following cholecystectomy.

## Acknowledgments

Thanks to Mr Xiaoyu Zhang for his outstanding contribution to this article.

## Author contributions

**Writing – original draft:** Yulai Yin.

**Writing – review & editing:** Xiaoyu Zhang.

## Supplementary Material


